# *In Silico* Design of Antimicrobial Peptides against Carbapenem-Resistant Infections with Enhanced Activity by Nanoformulation

**DOI:** 10.1021/acsabm.5c00679

**Published:** 2025-06-25

**Authors:** Lawrance Richardson, Tsung-Ying Yang, Yu-Wei Chen, Shang-Yi Lin, Yeng-Tseng Wang, Po-Liang Lu, Yang-Hsiang Chan, Hong-Cheu Lin

**Affiliations:** a Department of Materials Science and Engineering, 34914 National Yang Ming Chiao Tung University, Hsinchu 300, Taiwan; b Department of Medical Laboratory and Regenerative Medicine, 145474 MacKay Medical College, New Taipei City 252, Taiwan; c Research Institute for Science and Engineering, Waseda University, Tokyo 162-8480, Japan; d Department of Medical Laboratory Science and Biotechnology, College of Health Sciences, 38023 Kaohsiung Medical University, Kaohsiung 807, Taiwan; e Department of Medical Laboratory Science, College of Medical Science and Technology, 54791 I-Shou University, Kaohsiung 807, Taiwan; f Department of Internal Medicine, Kaohsiung Medical University Hospital, Kaohsiung Medical University, Kaohsiung 807, Taiwan; g School of Medicine, College of Medicine, 38023 Kaohsiung Medical University, Kaohsiung 807, Taiwan; h Department of Laboratory Medicine, Kaohsiung Medical University Hospital, Kaohsiung 807, Taiwan; i Department of Biochemistry, College of Medicine, 38023 Kaohsiung Medical University, Kaohsiung 807, Taiwan; j Center for Tropical Medicine and Infectious Disease Research, 38023 Kaohsiung Medical University, Kaohsiung 807, Taiwan; k Center for Liquid Biopsy and Cohort Research, 38023 Kaohsiung Medical University, Kaohsiung 807, Taiwan; l Department of Applied Chemistry, 34914 National Yang Ming Chiao Tung University, Hsinchu 300, Taiwan; m Center for Emergent Functional Matter Science, 34914 National Yang Ming Chiao Tung University, Hsinchu 300, Taiwan; n Department of Medicinal and Applied Chemistry, 38023 Kaohsiung Medical University, Kaohsiung 807, Taiwan

**Keywords:** antibiotic resistance, AMP, genetic algorithm, liposomal nanodelivery, biocompatibility

## Abstract

Carbapenem-resistant (CRAB) has emerged as a critical public health menace. Its resistance to last-resort antibiotics highlights the urgent need for innovative treatment approaches. Antimicrobial peptides (AMPs) are promising candidates to address this challenge. AMPs have distinct mechanisms and a low likelihood of inducing resistance. In this study, we designed a water-soluble cationic AMP, “T2–02.” This was achieved using AMP database screening and *in silico* modeling with genetic algorithms (GAs). T2–02 has a net +7 charge at physiological pH and is composed of 21 amino acid residues. This charge facilitates strong electrostatic interactions with negatively charged microbial membranes. Moreover, the helical secondary structure of T2–02 enhances amphipathicity, enabling effective membrane insertion. When tested against Gram-negative CRAB isolates, T2–02 showed strong antibacterial activity. It also demonstrated outstanding biocompatibility, with low cytotoxicity and a minimal inhibitory concentration (MIC) of 8–16 μg/mL. Its therapeutic potential was further enhanced by the use of a liposomal nanodelivery method. This significantly improved T2–02’s loading efficiency. The liposomal strategy amplified its antimicrobial efficacy, reducing MICs by 2- to 4-fold. It also further minimized cytotoxicity. These results position T2–02 as a promising candidate for combating CRAB infections.

## Graphical abstract

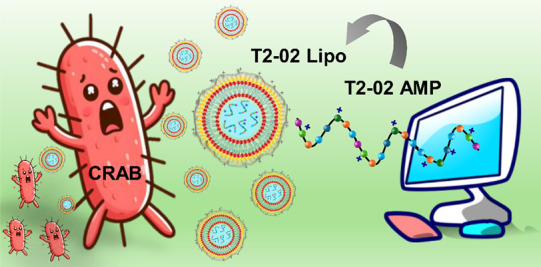

## Introduction

*Acinetobacter baumannii*, a Gram-negative pathogen, has emerged as a severe threat to human health worldwide particularly in immunocompromised individuals, and are linked to ventilator-associated pneumonia, bloodstream infections, and surgical site infections.^1^ Carbapenems such as imipenem and Meropenem are commonly used to treat *A. baumannii* infections but are increasingly compromised by resistance.^2^ Antibiotic resistance in *A. baumannii* increases rapidly each year, with Carbapenem-resistant *A. baumannii* (CRAB) posing the most significant challenges in healthcare settings. Based on the WHO’s 2018 list of ‘critical priority’ pathogens, CRAB can resist nearly all available antibiotics.^3^ Over the last two decades, only two antibiotic classes, oxazolidinones and cyclic lipopeptides, have been introduced, while both of which are effective only against Gram-positive bacteria.^4^ Alarmingly, infections caused by Gram-negative pathogens have been linked to 70–80% mortality rates.^5^ To address this challenge, there is an urgent need to explore alternative treatments and help combat antimicrobial resistance, particularly against Gram-negative bacteria.

Antimicrobial peptides (AMPs) represent a promising new class of alternatives to traditional antibiotics. AMPs are naturally abundant and play an important role in the innate immune system, which display a wide range of structures and functions.^6^ To date, more than 3,000 AMPs have been discovered but only seven of them have been approved by the U.S. Food and Drug Administration (FDA).^7^ One notable example is colistin (polymyxin E) which is used as a last-option treatment for multidrug-resistant Gram-negative bacterial infections, despite its associated risks of neurotoxicity and nephrotoxicity.^8^ This highlights the need for alternative antimicrobial strategies. AMPs, while promising, face significant challenges such as instability under environmental conditions (e.g., pH, temperature, UV exposure) and susceptibility to protease degradation.^9,10^ Accordingly, developing a delivery system which is highly effective, stable, and biocompatible is critical for addressing these issues and advancing AMP-based therapies against resistant bacterial infections.

The primary focus in designing AMPs is to target bacterial membranes by enhancing cationic charge and amphipathicity. This strengthen the interactions with negatively charged bacterial outer membrane components, such as lipopolysaccharides (LPS).^11,12^ The membrane is damaged by these interactions, which results in cytoplasmic leakage and cell death.^13,14^ AMPs are typically small (<10 kDa) and classified by secondary structures (α-helical, β-sheet, or extended) with α-helical structures primarily contributing toward amphipathicity.^15,16^ A high isoelectric point (pI > 8) could ensure AMPs remain positively charged at physiological pH. However, designing AMPs with optimal properties is challenging due to the vast number of possible sequence variants (X^20^, where X is the amino acids count in the peptide sequence), making exhaustive searches impractical. Traditional experimental methods face limitations in terms of stability, toxicity, time consumption and high production costs, which hinder their broader clinical application.^7,17^ Therefore, the introduction of computer-aided methods started to emerge in the related field.

Computer-aided “*in silico*” design approaches have become an attractive strategy for developing synthetic AMPs, offering a powerful alternative to traditional experimental methods.^18^ These approaches leverage data from AMP sequence databases (e.g., Collection of Antimicrobial Peptides (CAMPR3)) to guide the design of novel peptide sequences.^19^ Genetic algorithms (GAs) can be used for AMP generation by mimicking natural evolution. They begin with a population of random peptide sequences, evaluate their fitness (e.g., antimicrobial activity, toxicity), and iteratively apply selection, crossover, and mutation to evolve better candidates.^20^ Over generations, GAs optimize AMPs for desired properties such as efficacy against pathogens, toxicity, or stability, providing a computational approach to designing therapeutic peptides. This *in silico* approach for searching potential AMPs has been approved to be much easier, faster, and more cost-effective.^21,22^

Aiming at developing delivery systems of AMPs to further improve their therapeutic effectiveness, we also design formulation methods like encapsulation in liposomes^23–25^ and coacervates.^26^ While coacervates require additional steps to achieve nanosized formulations, liposomes are inherently nanosized (50–200 nm). Liposomes offer advantages like ease of surface modification, superior stability, and the capacity to encapsulate both hydrophilic and hydrophobic AMPs.^25–28^ As such, a direct comparison of their performance is essential to determine the most effective delivery system for improved therapeutic outcomes.

To summarize, two key limitations need to be addressed to advance the clinical use of AMPs: 1) Designing AMPs with minimal toxicity while maintaining high antimicrobial efficacy. 2) Developing suitable delivery systems to enhance stability, efficacy, bioavailability, and targeted delivery for improved therapeutic outcomes. In this study, we employed *in silico* design methods, leveraging GAs and AMP sequence databases to generate synthetic cationic AMPs. Starting with seq ID-1 AMP as a template^29^—a peptide effective as an environmental disinfectant but unsuitable for medical use due to its potent toxicity, we designed a series of peptides with enhanced antimicrobial activity. Among these, T2–02 demonstrate the superior antimicrobial performance. Attempting to enhance the therapeutic potential of T2–02, we further explored two delivery strategies: liposome (T2–02 Lipo) and coacervation (T2–02 coacervates)-based systems for comparison of antimicrobial activity.

## Results and Discussion

### Computational Training and Structural Modeling of Peptides

As shown in Scheme 1, we developed a computer-based method to design improved AMPs using three key components: 1) Molecular features, 2) Genetic algorithms, and 3) An antimicrobial scoring system. The genetic algorithm worked by evolving better peptide sequences from an initial set of 280 peptide sequences (length <30 amino acids) extracted from a computational database. (**See methods**) Our fitness function prioritized two key physiochemical properties - high pI and α helix propensity. These features were chosen because they help peptides interact with and disrupt bacterial membranes, as seen in natural AMPs like magainin’s^30^ and LL-37.^31^ The genetic algorithm carefully balanced two needs: exploring new sequence possibilities while maintaining useful features. It achieved this through frequent mixing of good sequences (0.8–1.0 crossover rate) with minimal random changes. We also ensured novelty by rejecting designs too similar (>40%) to existing patented peptides. The antimicrobial scoring system then helped identify the most promising candidates by evaluating their amino acid patterns. The analysis revealed that effective AMPs typically show high helical content (>40%) and avoid excessive coil or sheet structures (Figure 1). The results suggest that helical peptides can better penetrate microbial membranes, while too many coils or sheets might reduce their stability or targeting ability.^16,32^ We then set strict selection criteria, requiring designed peptides to exceed average natural AMPs in both helicity (≥48%) and positive charge (pI ≥ 9.63). These optimized selection criteria enabled us to computationally identify 11 promising AMP candidates (T1-T9, T2–01 and T2–02) whose physicochemical properties are detailed in Table 1. The Candidate peptides were further screened using the antimicrobial index (AMI) developed by Torrent et al.,^33^ which assigns coefficient values to amino acids based on their distribution in known AMPs (lower values indicating higher antimicrobial potential). (**see methods**) Initial computational screening yielded nine candidates (T1-T9), with T2 (ALWKDILKNAGKAALNEINQL) demonstrating superior experimental activity. Subsequent optimization of T2 produced additional candidates, including T2–02, with improved theoretical properties (antimicrobial index: 0.198, pI: 12.18) through strategic amino acid substitutions that enhanced antimicrobial characteristics while maintaining structural integrity. Its optimized sequence appears to maintain the ideal helical structure with increasing membrane-targeting capability. We then performed *in vitro* testing to verify whether the predicted pI increase (12.18) could improve antimicrobial activity or escalate hemolytic risk.

**Scheme 1:**
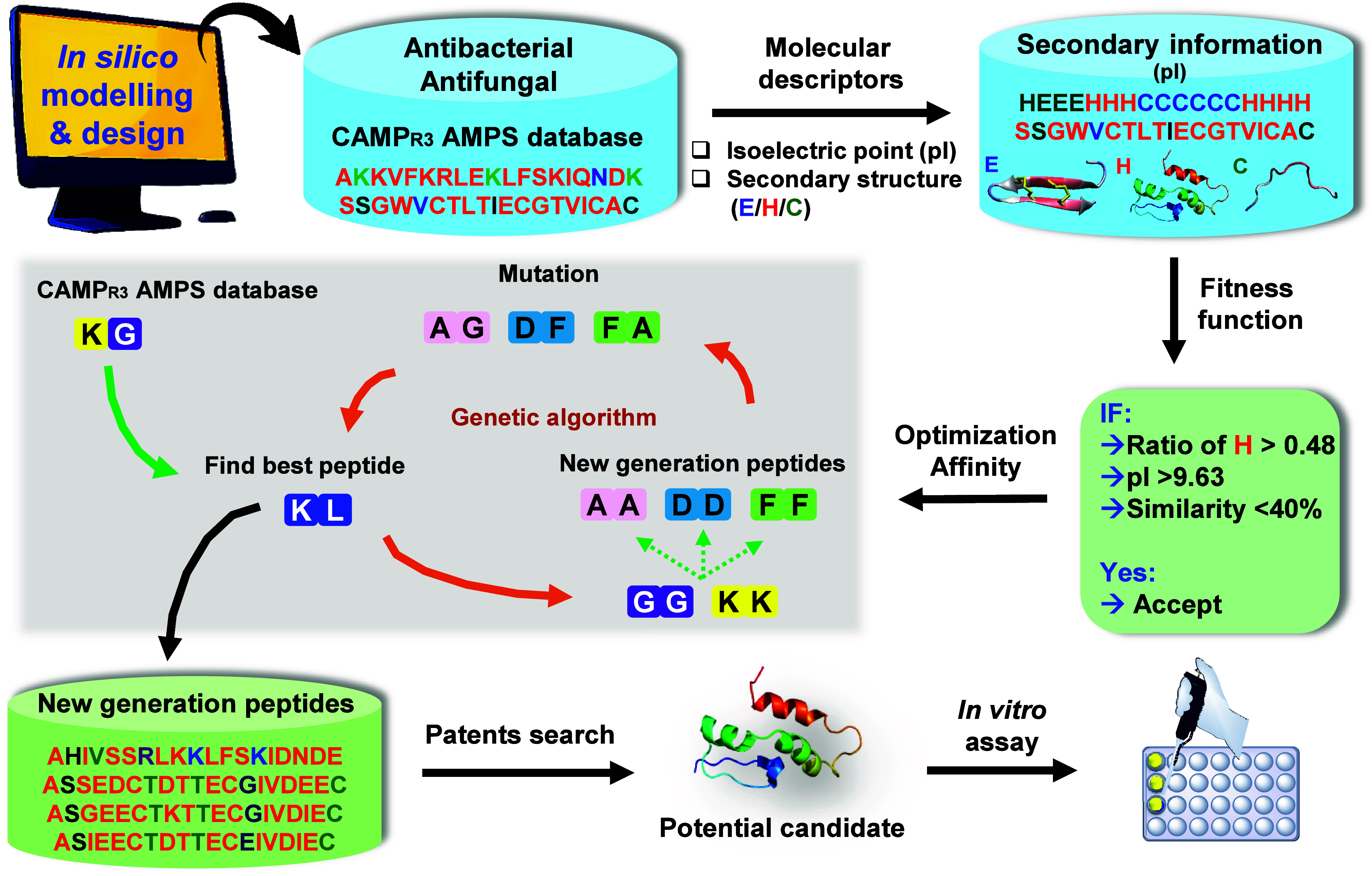
Schematic Representation of *In Silico* Design of Antimicrobial Peptides Using Antimicrobial Peptide Database and Genetic Algorithm

**Figure 1:**
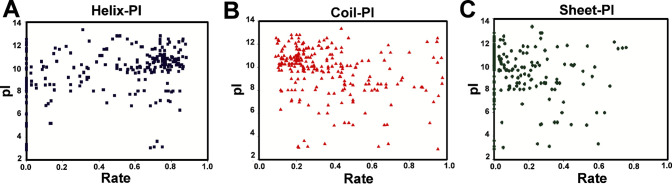
Fitness function plot of isoelectric point vs rate of (A) helix, (B) coil, and (C) Sheet formation for AMPs.

**Table 1:** Biophysical Data of the Peptides Utilized in This Study

Peptide name	Sequence	Net charge^a^	% Hydrophobicity^a^	% helix	pI^a^	AMI
**seq ID-1**	ALWKDILKNAGKAALNEINQL**VNGRGLKK**	+4	45	67	10.17	0.23
**T1**	VNGRGLKK	+3	25	0	11.17	0.23
**T2**	ALWKDILKNAGKAALNEINQL	+1	52	90	8.54	0.249
**T3**	LKNAGKAALNEINQLVNGRGLKK	+4	39	-	10.46	0.235
**T4**	LNEINQLVNGRGLKK	+2	33	57	9.90	0.235
**T5**	KNAGKAALNEINQLVNGRGLKK	+4	36	61	10.46	0.235
**T6**	KNGGKGGLNEINQLVNGRGLKK	+4	23	42	10.46	0.230
**T7**	KNCGKGGLNEINQLVNGRGLKK	+4	27	38	10.03	0.226
**T8**	KNCGKAALNEINQLVNGRGLKK	+4	36	66	10.03	0.229
**T9**	WKDILKNGGGAALNEINQLVNGRGLKK	+3	37	73	10.00	0.24
**T2–01**	ALWKRLLKRRGKAALNEINQL	+5	48	60	11.73	0.217
**T2–02**	ALWKRLLKRRGKIILNERLRL	+7	48	60	12.18	0.198

a Obtained using peptide-calculator server (https://www.bachem.com/knowledge-center/peptide-calculator/).

### *In Vitro* Antimicrobial Efficacy of AMPs

The results of antimicrobial efficacy (Table 2) show that the seq ID-1 and T2–02 peptides exhibit excellent antibacterial activities against key Gram-negative pathogens. Compared to T2 and T2–01 peptides, T2–02 exhibited more potent efficacy (minimal inhibitory concentration, MIC) against *E. coli* (MIC: 64 μg/mL), *K. pneumoniae* (128 μg/mL), *A. baumannii* (8 μg/mL), and *P. aeruginosa* (8 μg/mL). While T2–02’s activities against Gram-positive bacteria are also phenomenal compared to the other peptides (MIC: 64–256 μg/mL). Given T2–02’s exceptional potency against *A. baumannii*, we focus further research on CRAB clinical isolates, which are one of the major concerns in clinical settings due to their extensive resistance to available treatments. A consistent and potent antibacterial activity was revealed for T2–02 against 24 CRAB isolates (AB-01 to AB-24) with MIC values ranging between 8 and 16 μg/mL (vide infra). The micrographs captured by scanning electron microscopy (SEM) demonstrate that T2–02 at 8 μg/mL can notably affect the outer membrane of *A. baumannii* ATCC 19606, causing pore formation, collapse, and lysis (Figure 3, **i-iv**). The outstanding antibacterial activity of T2–02 can be attributed to its amphipathic structure and physicochemical properties. The α-helical structure of T2–02 segregates hydrophobic and hydrophilic residues into distinct faces, creating an amphipathic architecture. This enables the hydrophobic face to insert into the lipid bilayer and the positively charged face to interact electrostatically with bacterial membranes that are negatively charged.^14,34^

**Table 2:** MICs (in μg/mL) of Antimicrobial Peptide Analogues Tested against Various Gram-Positive and Gram-Negative Bacterium

Bacterial strains	seq ID-1	T2	T2–01	T2–02
*E. coil* ATCC25922	64	>1024	>512	64
*K. pneumoniae* ATCCC700603	128	>512	>512	128
*K. pneumoniae* ATCC BAA-1705	128	>1024	>512	512
*E. faecalis* ATCC 29212	128	1024	>512	64
*S. aureus* ATCC25923	128	1024	>512	256
*S. aureus* ATCC29213	>512	>512	512	64
*S. aureus* ATCC700698	>512	>512	>512	256
*S. aureus* ATCC33592	>512	>512	>512	256
*A. baumanni***i** ATCC19606	32	8	64	8
*A. baumannii* ATCC BAA-747	32	8	64	8
*P. aeruginosa* ATCC27853	32	8	64	8

The T2–02 AMP sequence consists of 21 amino acid residues. Parts of the enhanced activity are due to its high net charge of +7 which primarily contributed by Lysine (K) and Arginine (R) residues. K contributes 3 units (14%) of the total charge, while R contributes 5 units (24%), providing a total of 8 cationic residues. However, R’s guanidinium group stabilized by resonance maintains a more stable positive charge. This enhanced stability enables R to participate more effectively in electrostatic interactions and structural roles within the peptide.^35^ Compared to K, R residues play a more prominent role in T2–02 as shown by their greater contribution compared to related peptides like seq ID-1 (R= 3%), T2 (R= 0%), and T2–01 (R= 14%). Therefore, the high positive charge of T2–02 boosts its capacity to engage in electrostatic interactions with bacterial membrane components, such as LPS that are negatively charged. These interactions enable the peptide to bind to the membrane initially and disrupt it subsequently, which is central to T2–02’s potent antibacterial activity.^14^

Following initial electrostatic attraction, T2–02’s hydrophobic domains—Isoleucine (I), Leucine (L), Alanine (A), and Tryptophan (W)—interact with the lipid bilayer, particularly in the LPS layer of Gram-negative bacterial membranes. Leucine and Isoleucine (with four-carbon hydrophobic side chains) strengthen these interactions by increasing membrane permeability and disrupting the barrier function of target pathogens.^36^ The helical conformation facilitates efficient membrane insertion and then leads to disruption or pore formation that is critical for bactericidal activity.^16^ Both T2–02 and T2–01 exhibit 60% helical content, while T2 has 90% helical content. Although they share similar hydrophobicity in the range of 45–52%, T2–02’s high isoelectric point (pI = 12.18) ensures its highly cationic at physiological pH (7.4). This can enhance electrostatic interactions with bacterial membranes. These combined properties, including high net positive charge, helical structure, and hydrophobicity, contribute to T2–02’s potent antibacterial activity and make it more effective than other peptide variants.

Despite its potent antibacterial activity, T2–02 exhibits certain limitations: The requirement of higher concentrations (MIC: 8–16 μg/mL) to effectively treat CRAB infections compared to conventional antibiotics like colistin (MIC: 0.5 μg/mL).^26,37^ This suggests a relatively lower antibacterial potency against CRAB isolates. Even so, T2–02’s safety profile is a significant advantage. It has been reported that high hydrophobicity (>40–60%) may cause nonspecific interactions with host cell membranes and thus induce cytotoxicity.^38,39^ In this work, the hydrophobicity of 48% for T2–02 balances antibacterial efficacy and low cytotoxicity. The high biocompatibility of T2–02 was approved by the cytotoxicity tests on HK-2 cells showing an IC_50_ value (i.e., the concentration required to cause 50% cell death) of 557 μg/mL for T2–02, which is 30.9, 11.6, and 2.6 times higher than the standard antibiotics polymyxin B (PMB),^40–42^ colistin,^26,37^ and SPR206,^41^ respectively (Table 3). These results indicate substantially lower toxicity and a better safety profile for T2–02 as a promising therapeutic candidate.

**Table 3:** Comparison of Cytotoxicity of Various Antimicrobial Compounds with Free T2-02 and T2-02 Lipo

	IC_50_ (μg/mL)				
Compound	HK-2	HEK293	THP-1	Cytotoxicity HK-2 related to PMB	ref.
Polymyxin B (PMB)	18	∼500	∼125	1	References 40–42
Colistin	46.8	>500	>250	2.6	References 26,27
SPR 206	208.8	NR	NR	11.6	Reference 41
seq.ID-1	NR	67.2	NR	-	Reference 29
T2	NR	>1024	NR	-	This study
T2–02	557	1101	866	30.9	This study
T2–02 Lipo	819.62	>1024	>1024	45.5	This study

The antimicrobial mechanism of colistin against bacteria has been attributed to the interaction between positively charged colistin and negatively charged outer membrane.^43^ Colistin could be displaced by divalent ions, such as calcium ions (Ca^2+^) and magnesium (Mg^2+^) in a competitively way.^43^ Moreover, elevated levels of divalent cations can enhance membrane rigidity, thereby reducing drug penetration and overall efficacy.^44^ To investigate the antimicrobial mechanism of T2–02, the competitive assays using divalent calcium ion were conducted. The elevated MICs of colistin were observed by adding Ca^2+^ (2 to 32 μg/mL, Table S4). Similar results were found for T2–02 where the MICs increased from 8 to over 256 μg/mL, implying a similar antimicrobial mechanism of T2–02 with colistin.

### Designing Strategy and Characterization of T2–02 Nano Formulations

To further improve the therapeutic potential of T2–02, we explored two encapsulation approaches: liposome-based delivery (T2–02 Lipo) and coacervation (T2–02 coacervates). By comparing the *in vitro* efficacy of these two nanoformulation strategies, we aimed to identify the most effective method for encapsulating the T2–02 AMP.

#### T2–02 Lipo

The thin-film hydration technique was used to prepare the ″T2–02 Lipo″ nanoparticles (NPs), as illustrated in Scheme 2. In this process, a phospholipid bilayer thin film composed of DOPC and DSPE-PEG2000 was hydrated with a T2–02 solution in deionized water, followed by vortex mixing at a weight ratio of 4:1:1 (T2–02: DOPC: DSPE-PEG2000). The resulting liposomal suspension was centrifuged to isolate the bulk residue containing T2–02-loaded liposomes, which were then diluted in deionized water to achieve the desired peptide concentration. To enhance colloidal stability, the liposomes were coated with high molecular weight poly-l-lysine (PLL, 30–70 kDa) at an optimal amount of 55 μg to ensure a zeta potential ≥ + 30 mV. However, we found that excessive PLL increased toxicity toward HK-2 cells, highlighting the need for careful optimization to balance stability and biocompatibility. Therefore, a series of seven formulations (Lipo-1, Lipo-2, Lipo-3, Lipo-4, Lipo-5, Lipo-6 and T2–02 Lipo) were prepared with different amounts of PLL used in the formulation as shown in Table S1. The DLS, TEM and Zeta potential measurements confirmed that liposomes remained colloidally stable only when coated with PLL because uncoated liposomes tended to aggregate and destabilize over time (Figure 2 **D,** Figure S2, Figure S3, Figure S4 and Figure S9). The optimum formulation is chosen by means of their IC_50_ cytotoxicity profile and MIC detection for varying amounts of PLL (Table S5). The successful coating of phospholipids and PLL was further validated by a stable charge reversal, as evidenced by zeta potential analysis (Figure 2C). The PLL coating on the liposome surface was also verified by transmission electron microscopy (TEM) imaging of T2–02 Lipo (**inset** in Figure 2B). Both dynamic light scattering (DLS) and TEM verified that the particle size ranged between 100 and 200 nm **(** Figure 2 **A and[Fig fig2]B)**. Encapsulation efficiency analysis demonstrated that increasing the initial T2–02 concentration by up to four times resulted in higher peptide loading within the liposomes. The optimized T2–02 Lipo formulation achieved an encapsulation efficiency of 63.4% (Table S2 **and see methods section**). The T2–02 Lipo NPs were further analyzed for their *in vitro* performance to evaluate their therapeutic potential and efficacy.

**Scheme 2:**
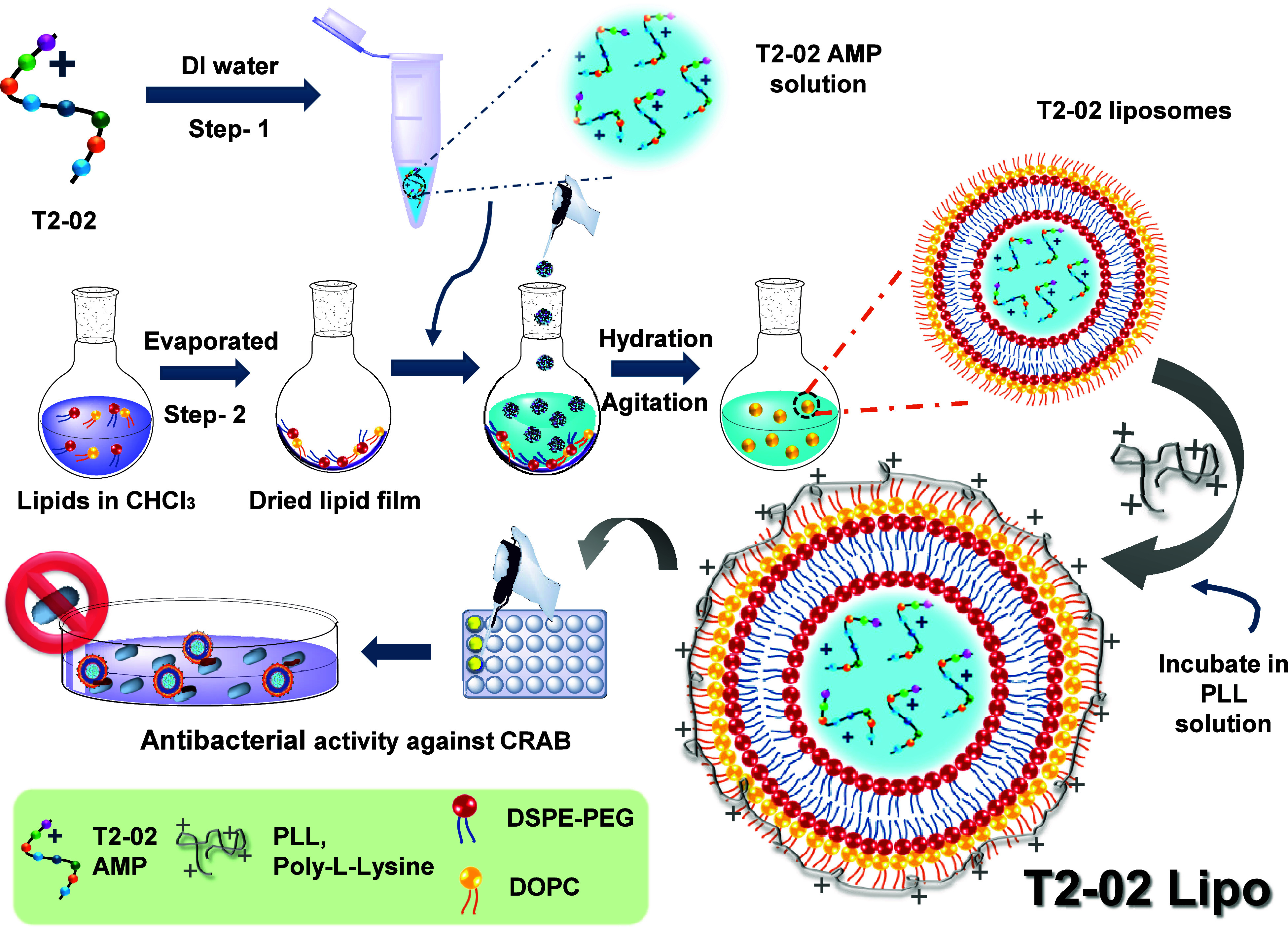
. Schematic Representation of Strategy to Construct T2-02 Lipo Nanoparticles against CRAB

**Figure 2:**
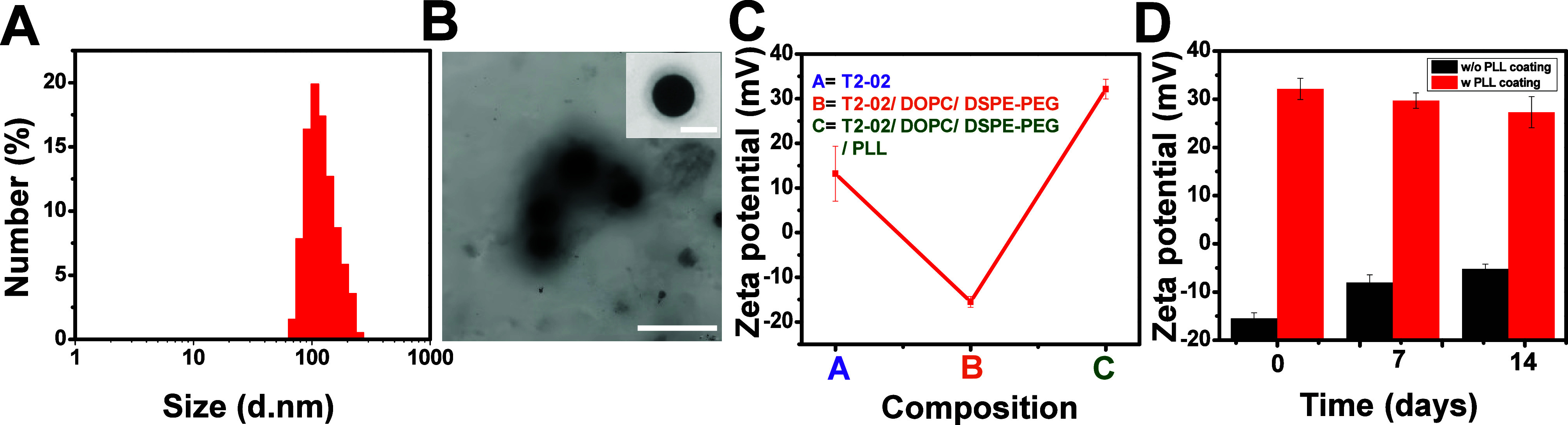
(A) Hydrodynamic diameter of T2–02 Lipo nanoparticles, measured by DLS, showing an average size of 131.9 ± 55 nm (*n* = 3). (B) TEM images of T2–02 Lipo nanoparticles (scale bar: 2 μm). The inset highlights a single T2–02 Lipo nanoparticle with a PLL coating on its surface (scale bar: 200 nm). (C) Zeta potential measurements demonstrating charge reversal upon T2–02 Lipo formation. (D) Zeta potential analysis indicating the colloidal stability of T2–02 Lipo nanoparticles with (w) and without (w/o) PLL coating. Statistical analyses were performed with a sample size of three (*n* = 3) measured at 25 °C.

#### T2–02 Coacervate NPs

We first performed a screening experiment by combining the cationic T2–02 AMP and the anionic Lauric acid (LA) in different weight ratios to induce coacervation between the two substances. There was no discernible phase separation for T2–02:LA weight ratios lower than 7:3 (Figure S5). However, when the T2–02:LA weight ratio exceeded 7:3, the solution turned cloudy, indicating the formation of liquid droplets “T2–02 coacervates” (Figure S5A **and inset in** Figure S5D). As the weight ratio increased from 7:3 to 9:1, the coacervates stabilized into a cloudy suspension. The formation of coacervates was observed using optical microscopy, and DLS analysis confirmed that the particle size was in the micron range (Figure S6A). Among these ratios, the 8:2 formulation demonstrated superior colloidal stability over time and was therefore selected for further steps. It is likely that the electrostatic interaction between LA and T2–02 led to the formation of such complex coacervates capable of phase separation due to surface hydrophobicity and electrostatic forces. However, the development of coacervate droplets as therapeutic delivery systems faces challenges due to their large size, inherent lack of physiological stability and absence of a membrane structure. To address these limitations, we coated the coacervate droplets following the liposomal strategy as shown in Scheme S1 with a PEGylated phospholipid membrane composed of DOPC and DSPE-PEG2000, followed by an additional layer of PLL to enhance colloidal stability and achieve a highly positive zeta potential (Figure S8). The successful coating with phospholipids was confirmed by the stable reversal of surface charge (Figure S7). The particle size of the resultant ″T2–02 coacervate NPs″ ranged from 100 to 200 nm (Figure S6B). The observed size reduction is consistent with previous reports showing that lipid vesicle coating, particularly with PEGylated lipids, promotes interfacial assembly, steric stabilization, and droplet compaction, resulting in smaller and more stable coacervates.^45,46^ The “T2–02 coacervate” and “T2–02 coacervate NPs” were subsequently evaluated *in vitro* against CRAB isolates to assess their therapeutic efficacy.

### *In Vitro* Efficacy of T2–02 Nanoformulations

The study evaluated the antibacterial efficacy and cytotoxicity of four liposomal formulations—Lipo-1, Lipo-2, Lipo-3, and T2–02 Lipo—alongside T2–02 coacervates and T2–02 coacervate NPs. T2–02 Lipo demonstrated superior performance compared to free T2–02, with a two- to 4-fold reduction in MIC values across 24 CRAB isolates (Table 4).

**Table 4:** MICs of Free T2-02 and T2-02 Lipo against Different Gram-Negative CRAB Isolates

CRAB isolates	Free T2–02 (μg/mL)	T2–02 Lipo (μg/mL)	Fold-decrease
**AB-01**	8	8	-
**AB-02**	8	2	4
**AB-03**	4	2	2
**AB-04**	8	4	2
**AB-05**	8	2	4
**AB-06**	8	4	2
**AB-07**	8	2	4
**AB-08**	8	16	-
**AB-09**	8	8	-
**AB-10**	8	8	-
**AB-11**	8	4	2
**AB-12**	16	4	4
**AB-13**	4	2	2
**AB-14**	8	2	4
**AB-15**	8	2	4
**AB-16**	16	4	4
**AB-17**	8	4	2
**AB-18**	16	4	4
**AB-19**	8	4	2
**AB-20**	8	4	2
**AB-21**	8	2	4
**AB-22**	8	2	4
**AB-23**	8	4	2
**AB-24**	8	8	-

This enhancement could be attributed to the liposomal structure, which features stable phospholipid bilayers that effectively interact with the Gram-negative bacterial membrane component, LPS. The cationic polymer PLL used in the formulation further enhances antibacterial efficacy by interacting with negatively charged bacterial membranes, increasing drug accumulation at bacterial sites, and protecting the antibiotic from degradation. However, higher PLL concentrations increase cytotoxicity as evidenced by an IC_50_ of 23 μg/mL for HK-2 cells in Lipo-1. Reducing PLL to 0.055 mg in Lipo-3 minimized toxicity with an IC_50_ of 514 μg/mL, which is comparable to free T2–02 (557 μg/mL). We further optimized the formulation by increasing the amounts of T2–02 AMP to produce T2–02 Lipo with an encapsulation efficiency of 63.41%. T2–02 Lipo showed a significant reduction in cytotoxicity, yielding an IC_50_ of 819.62 μg/mL for HK-2 cells. These results highlight the ability of T2–02 Lipo to lower cytotoxicity while enhancing antibacterial efficacy.

In contrast, T2–02 coacervates exhibited low antimicrobial performance with MIC values exceeding 32 μg/mL across 13 CRAB isolates (Table S6). This inefficiency is likely due to their larger size limiting bacterial membrane permeability as well as their poor stability under physiological conditions including salt induced aggregation (Figure S10). Downsizing T2–02 coacervates into NPs, T2–02 coacervate NPs (100–200 nm) improved their MIC values to 8–16 μg/mL, comparable to free T2–02 (Table S6). However, these coacervate NPs still showed high cytotoxicity with an IC_50_ of 15 μg/mL for HK-2 cells, likely due to the higher PLL concentrations required (Table S3). This suggests that PLL does not significantly contribute to antibacterial activity but rather exacerbates toxicity at elevated levels. The low performance of coacervates suggests their limitations as a delivery system compared to liposomes.

To assess the antibacterial mechanism of T2–02 Lipo, SEM and time-kill curves were conducted. The micrographs showed morphological changes in ATCC 19606 isolate following treatment with increasing concentrations (8, 16, and 32 μg/mL), revealing dose-dependent membrane damage with surface leakage, pronounced wrinkling, and eventual cell lysis (Figure 3). These structural deformations suggested that T2–02 Lipo exerted its bactericidal effect through direct damage to the bacterial cell envelope. Time-kill assays against CRAB ATCC 19606 showed that T2–02 Lipo achieved complete bacterial eradication (>99.9%) at all tested concentrations we tested (8, 16, and 32 μg/mL) following 24-h incubation. Compared to free T2–02, the curves of T2–02 Lipo exhibited more quick-act antibacterial activities. At 8 μg/mL, T2–02 Lipo demonstrated more remarkable eradication with only 90% reduction found for free T2–02. (Figure 4). To assess red blood cell (RBC) compatibility, a hemolysis assay was conducted for free T2–02, colistin, and T2–02 Lipo over a concentration range of 0–256 μg/mL. Free T2–02 exhibited high hemolytic activity (∼100% at 256 μg/mL), while colistin showed moderate hemolysis (∼45% at 256 μg/mL). Notably, T2–02 liposomes induced significantly lower hemolysis (<40%), indicating that encapsulation and surface modification with PLL mitigates the cytotoxic effect on RBCs (Figure 5). The findings demonstrate that liposomal encapsulation significantly enhances the therapeutic efficacy and safety of T2–02, offering a broader therapeutic window by reducing cytotoxic effects.

**Figure 3:**
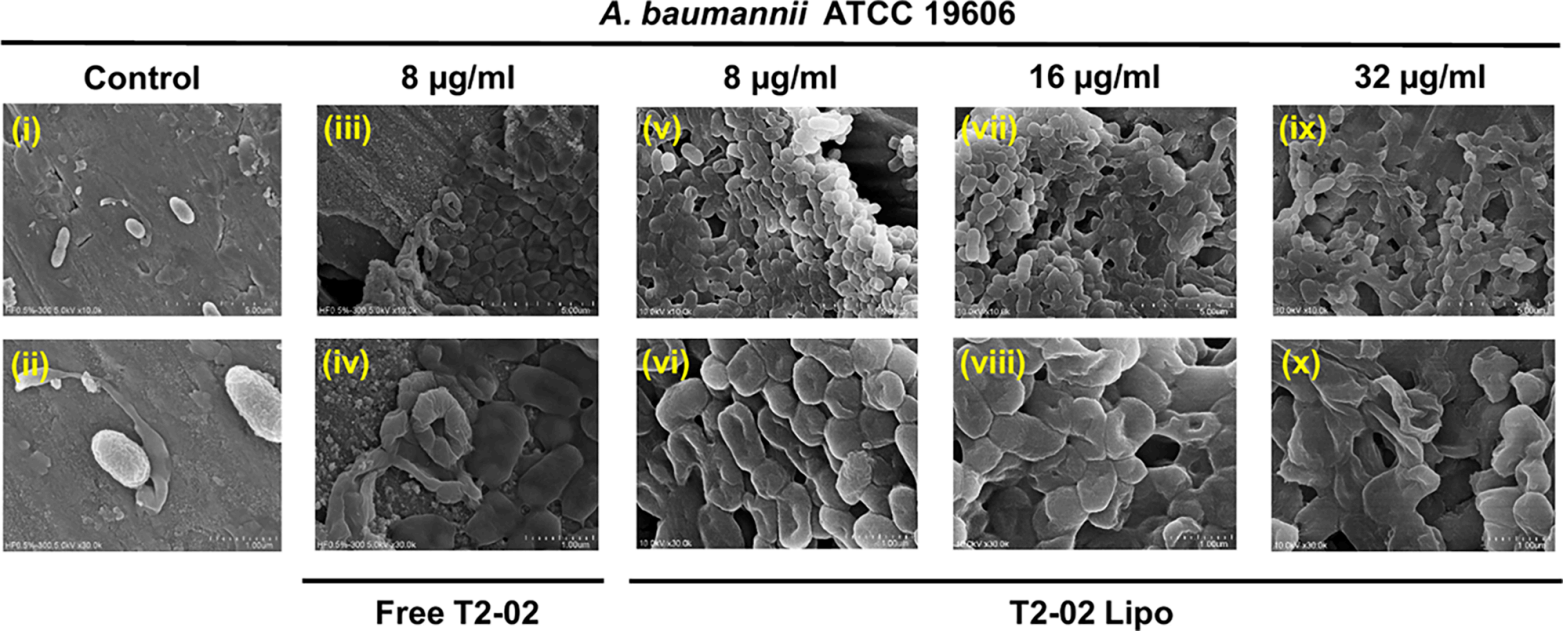
. SEM images of ATCC 19606 bacteria treated with Free T2–02 and T2–02 Lipo, captured at 10,000× (i, iii, v, vii, ix) and 30,000× magnification (ii, iv, vi, viii, x). Untreated bacteria (i, (ii) exhibited a smooth and intact morphology. In contrast, bacteria treated with 8 μg/mL of Free T2–02 and (8, 16, and 32 μg/mL) of T2–02 Lipo showed membrane lysis and porous structures (shown in iv, vi, viii and x).

**Figure 4:**
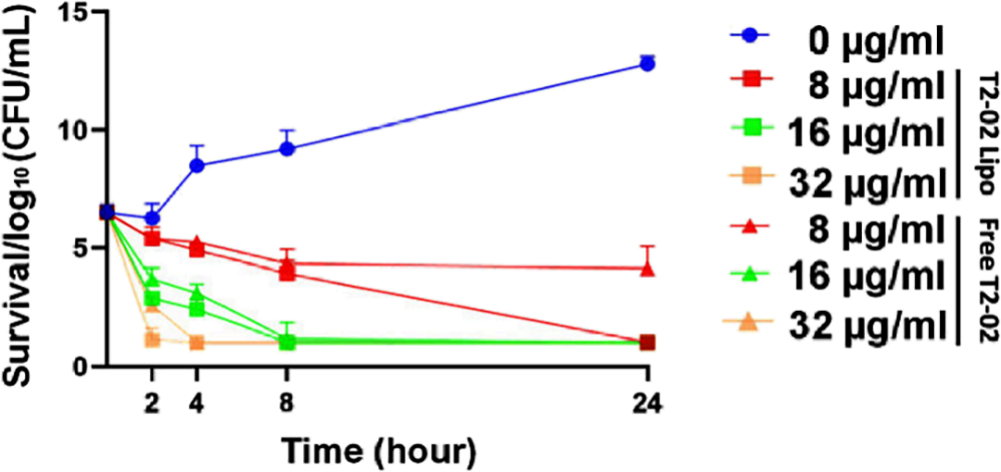
Time-kill kinetic assay of Free T2–02 and T2–02 Lipo against CRAB ATCC 19606.

**Figure 5:**
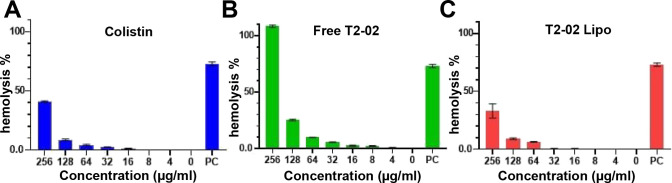
. Hemolysis profiles of colistin, free T2–02, and T2–02-loaded liposomes. (A) Colistin, (B) free T2–02 peptide, and (C) T2–02-loaded liposomes (T2–02 Lipo) were incubated with human red blood cells (RBCs) at various concentrations (0–256 μg/mL) for 1 hour at 37 °C.

## Conclusions

In conclusion, we have successfully designed and optimized the cationic AMP T2–02 using computational modeling by leveraging AMP database screening and genetic algorithms. T2–02 demonstrated potent activity against CRAB isolates, high biocompatibility, and low cytotoxicity. Two encapsulation strategies: coacervation and liposome-based delivery were explored and compared. T2–02 coacervates showed modest efficacy similar to the free peptide. On the other hand, liposomal encapsulation with PLL surface modified positively charged liposomes significantly enhanced *in vitro* antimicrobial activity, reduced the MIC by 2–4 times, and significantly lowered cytotoxicity. These findings were validated through electron microscopy and time-kill curves to confirm rapid and sustained bacterial killing. The liposomal delivery system proved superior, highlighting its potential to optimize T2–02’s therapeutic performance. Building on these promising *in vitro* results, future work will focus on evaluating the *in vivo* efficacy of T2–02-loaded liposomes in preclinical models, advancing its potential as a next-generation antimicrobial therapeutic for clinical translation against CRAB infections.

## Experimental Section

### Materials

The blood samples of patients from Kaohsiung Medical University were used to collect and cryopreserve all of the CRAB clinical isolates.^47^ Analytical graded chemicals and reagents were utilized throughout the investigation. The T2–02 peptide (MW 2646.27 Da) was custom-synthesized by Kelowna International Scientific Inc., 1,2-Dimyristoyl-*sn*-glycero-3-phosphoethanolamine-N-[methoxy­(polyethylene glycol)-2000] (ammonium salt) (DSPE-PEG2000, MW 2693.285 Da) was purchased from Laysan Bio Inc., 1,2-Dioleoyl-*sn*-glycero-3-phosphocholine (DOPC, MW 786.1 Da) was acquired from BroadPharm, and Poly-l-lysine hydrobromide (30–70 kDa) was sourced from Sigma-Aldrich.

### Methods

#### Computational Tools and Parameters

Antimicrobial peptides (AMPs) were optimized using a computational approach combining molecular descriptors, genetic algorithms, and antimicrobial index screening. A data set of 280 peptide sequences (length <30 amino acids) with activity against Gram-positive bacteria, Gram-negative bacteria, and fungi was extracted from the CAMPR3 database.^48^ Molecular descriptors were calculated using IPC software^49^ for isoelectric point (pI) determination and PSIPRED^49^ for secondary structure prediction, with the latter employing neural networks trained on evolutionary information from PSI-BLAST (accuracy ∼ 80%). Analysis revealed that >60% of effective AMPs possessed Helix proportions >0.4, Coil and Sheet proportions <0.5, and pI values >8.0. A fitness function was established requiring candidates to exceed mean training set values (Helix ≥ 0.48, pI ≥ 9.63) and demonstrate <40% similarity to USPTO database sequences. The genetic algorithm^50^ implementation performed selection operations (calculated via λ=Φ/ΣΦ and ep = λ·P equations), crossover operations (probability 0.8–1.0), and mutation operations (minimal probability) to prevent premature convergence to local optima.

#### Antimicrobial Index (AMI) Calculation

The antimicrobial potential of the peptides were evaluated using the statistically validated AMI scoring system grounded by Torrent et al.^33^ This model derives residue-specific coefficients from >1,700 AMPs, where lower AMI values (<0.225) correlate with higher antimicrobial activity. The AMI scores for each peptide were calculated based on amino acid frequencies, with T2–02 (AMI = 0.198) falling below the predictive cutoff for antimicrobial regions, unlike suboptimal variants (e.g., T2: AMI = 0.249). For visual mapping of antimicrobial regions, the AMPA server (https://tcoffee.crg.eu/apps/ampa/guide.html) was employed.

#### Preparation of T2–02 Liposomes (T2–02 Lipo)

The thin-film hydration technique was employed to prepare T2–02 Lipo. In a round-bottom flask, 1.1 mg of DOPC and 1.1 mg of DSPE-PEG2000 were first dissolved in chloroform in a 1:1 weight ratio. After removing the solvent by rotary evaporation under vacuum for half an hour, any remaining chloroform was eliminated by nitrogen flushing. Now, a 4.4 mg T2–02 AMP solution in 1 mL deionized (DI) water was added right away to the flask, vortexed for 60 s at 1000 rpm, and then hydrated for 10 min. A centrifuge tube was then filled with 1 mL of the liposomal solution, left for an hour, then centrifuged for 15 min at 10,000 rpm. After separating the residue from the supernatant, the residue was resuspended in 0.4 mL of DI water and vortexed for 1 min. A 0.1 mL solution containing 55 μg of poly-l-lysine (PLL, 30–70 kDa) was gently mixed with the diluted liposomal suspension to complete the synthesis of T2–02 Lipo nanoparticles. The mixture was then allowed to sit at room temperature for 30 min.

### Characterization Techniques

#### Particle Size and Zeta Potential

At 25 °C, the average particle size and zeta potential were measured using a Malvern Nano-ZS90 dynamic light scattering (DLS) analyzer. Transmission electron microscopy (TEM) images of the generated liposomes were captured using a JEOL 2100 TEM instrument running at a 200 kV acceleration voltage. To do TEM analysis, a drop of the liposomal aqueous solution was applied to a copper mesh and dried at room temperature.

#### Encapsulation efficiency

The water-soluble unencapsulated T2–02, along with phospholipids, remained in the supernatant. The absorbance of the mixture, supernatant (unencapsulated T2–02), and residue (encapsulated T2–02) at 280 nm were measured using a UV–vis spectrophotometer in order to evaluate the EE. The absorbance of the mixture was subtracted from the absorbance of the supernatant to find the absorbance of the residue. To quantify the amount of T2–02 contained in the liposomes, a standard calibration curve plotting absorbance against known concentrations of the T2–02 peptide was drawn. (Figure S1) The encapsulation efficiency was calculated as follows:

$$(\mathrm{Encapsulation}\;\mathrm{efficiency})\%=\frac{\mathrm{Actual}\;\mathrm{amount}\;\mathrm{of}\;T2-02\;\mathrm{loaded}\;\mathrm{in}\;\mathrm{liposomes}}{\mathrm{Actual}\;\mathrm{amount}\;\mathrm{of}\;T2-02\;\mathrm{used}\;\mathrm{for}\;\mathrm{liposomal}\;\mathrm{preparation}}\times 100$$

#### Minimum Inhibitory Concentration (MIC)

The broth microdilution procedure involves preparing CAMHB broth, a bacterial suspension adjusted to McFarland 0.5 (1.5 × 10^8^ CFU/mL) and diluted 200×, and a drug solution at 2× the final target concentration. To achieve a 400× final bacterial dilution, add 100 μL of the drug at different doses to a 96-well plate, then 100 μL of the diluted bacterial culture. Measure the initial absorbance (0 h), incubate at 37 °C for 16–18 h, and measure the final absorbance. With no change in absorbance, the MIC signifies the absence of bacterial growth.

#### Time-Kill Assays

A previously established procedure was followed for performing time-kill tests.^47^ Briefly, At 37 °C, the bacterial strain ATCC 19606 was treated with 1×, 2×, and 4× MIC of T2–02 and several nano formulations, along with a 5% ethanol control, after being adjusted to 10^6^ CFU/mL in BHI broth. At certain periods of time (0, 2, 4, 8, and 24 h), bacterial populations were measured. A serial 10-fold dilutions prepared in 1× PBS were used to plate LB agar. Following 18 h of culture at 37 °C, colonies ranging from 25 to 250 were enumerated. As controls for bactericidal and bacteriostatic effects, respectively, rifampin and minocycline were employed.

#### Hemolysis Assay

The hemolytic potential of colistin, T2–02, and T2–02 Lipo were assessed using a modified erythrocyte lysis assay.^51^ Briefly, fresh erythrocytes were washed twice with phosphate-buffered saline (PBS) and resuspended in 5% glucose solution to prepare a 2% hematocrit suspension. Aliquots (100 μL) of each test compound at varying concentrations were incubated with the erythrocyte suspension at 37 °C with gentle agitation (120 rpm) for 1 h. After incubation, samples were centrifuged at 700 × g for 10 min, and the supernatant was transferred to a 96-well microplate. The release of hemoglobin was quantified by measuring the absorbance at 540 nm. The percentage of hemolysis was calculated as follows: Hemolysis (%) = (OD of test sample – OD of negative control)/(OD of positive control-OD of negative control) × 100%. Each experiment was performed in triplicate, and data were expressed as mean ± standard deviation (SD).

#### Electron Microscopy

The effect of T2–02 and T2–02 Lipo’s impact on bacteria’s morphology was studied by scanning electron microscopy (SEM). T2–02/T2–02 Lipo was introduced to *A. baumannii* ATCC 19606 bacterial cells for 1 h before to collection at varying doses of 8, 16, and 32 μg/mL. The procedure described in a previous study was followed in the preparation of the samples.^47^

#### Chemical Mechanism Study- Pharmacological Manipulation

As previously reported,^47^ the MIC values of T2–02 in conjunction with different chemical agents was estimated using the broth microdilution technique. Different concentrations of T2–02 were combined with Ca2+ separately, and the effect of chemical agents on T2–02 were tracked by observing variations in their MIC values. Each solution was added to a 96-well plate at the proper concentration in 100 μL volume. Next, 100 μL of the modified bacterial solution was added to each well. The absorbance was measured after incubation at 37 °C for 0 and 18 h.

#### Cytotoxicity Assay

We tested the cytotoxicity of several antibiotic compounds, including peptides and nanoparticles, on the human embryonic kidney cell lines HEK-293, HK-2, and NIH 3T3. The cells were stored in DMEM medium and incubated at 37 °C with 5% CO2 in a humidifying environment. Using the MTT (methylthiazolyldiphenyl-tetrazolium bromide) test, cell viability was evaluated. Following a 5 × 10^3^ cell/mL seeding in 96-well plates, the cells were exposed to 100 μL of samples at varying concentrations (12.5–200 μM) during 72 h at 37 °C. Later, each well is loaded with MTT solution (5 mg/mL in PBS) at a volume of 10 μL. The plates were left in the dark for 4 h. After forming formazan crystals due to the action of mitochondrial reductases in living cells, the crystals were dissolved using a solubilizing solution (pH 4.7, 40% dimethylformamide, 2% glacial acetic acid, and 16% sodium dodecyl sulfate) were shaken at 150 rpm and incubated for an hour at 37 °C. Using graphical plot analysis, the IC50 value—the concentration that results in 50% cell death—was obtained by measuring the absorbance of the dissolved formazan at 570 nm. The average of three independent tests, each carried out in triplicate, was used to display the results.

## Supplementary Material



## Notes

Additional experimental details, materials, and methods including photochemical characterizations including UV, DLS, TEM images, and coacervate microscope images; MIC and cytotoxicity data; and zeta potentials of nanoparticles (PDF)
