# Supplementary material for: In Silico Design of Antimicrobial Peptides against Carbapenem-Resistant Infections with Enhanced Activity by Nanoformulation
Source: ACS Appl Bio Mater. 2025 Jun 25;8(7):6121–31. doi: 10.1021/acsabm.5c00679 (PMC12284850; doi:10.1021/acsabm.5c00679)
Supplement: Supplementary file 1 [file mt5c00679_si_001.pdf]

Supporting Information

***In Silico* Design of Antimicrobial Peptides Against Carbapenem-Resistant *Acinetobacter baumannii* Infections with Enhanced Activity by Nano-Formulation**

Lawrance Richardson,[a†] Tsung-Ying Yang,[bcd†] Yu-Wei Chen,[e] Shang-Yi Lin[fgh], Yeng-Tseng Wang,*[i] Po-Liang Lu,*[jklm] Yang-Hsiang Chan,*[nop] Hong-Cheu Lin*[qr]

[a]*Department of Materials Science and Engineering, National Yang Ming Chiao Tung University, Hsinchu 300, Taiwan.*

[b]*Department of Medical Laboratory and Regenerative Medicine, MacKay Medical College, New Taipei City 252, Taiwan.*

[c]*Research Institute for Science and Engineering, Waseda University, Tokyo 162-8480, Japan.*

[d]*Department of Medical Laboratory Science and Biotechnology, College of Health Sciences, Kaohsiung Medical University, Kaohsiung 807, Taiwan.*

[e]*Department of Medical Laboratory Science, College of Medical Science and Technology, I-Shou University, Kaohsiung 807, Taiwan.*

[f]*Department of Internal Medicine, Kaohsiung Medical University Hospital, Kaohsiung Medical University, Kaohsiung 807, Taiwan.*

[g]*School of Medicine, College of Medicine, Kaohsiung Medical University, Kaohsiung 807, Taiwan.*

[h]*Department of Laboratory Medicine, Kaohsiung Medical University Hospital, Kaohsiung 807, Taiwan.*

[i]*Department of Biochemistry, College of Medicine, Kaohsiung Medical University, Kaohsiung 807, Taiwan*

[j]*Department of Internal Medicine, Kaohsiung Medical University Hospital, Kaohsiung Medical University, Kaohsiung 807, Taiwan.*

[k]*School of Medicine, College of Medicine, Kaohsiung Medical University, Kaohsiung 807, Taiwan.*

[l]*Center for Tropical Medicine and Infectious Disease Research, Kaohsiung Medical University, Kaohsiung 807, Taiwan.*

[m]*Center for Liquid Biopsy and Cohort Research, Kaohsiung Medical University, Kaohsiung 807, Taiwan.*

[n]*Department of Applied Chemistry, National Yang Ming Chiao Tung University, Hsinchu 300, Taiwan.*

[o]*Center for Emergent Functional Matter Science, National Yang Ming Chiao Tung University, Hsinchu 300, Taiwan.*

[p]*Department of Medicinal and Applied Chemistry, Kaohsiung Medical University, Kaohsiung 807, Taiwan.*

[q]*Department of Materials Science and Engineering, National Yang Ming Chiao Tung University, Hsinchu 300, Taiwan.*

[r]*Center for Emergent Functional Matter Science, National Yang Ming Chiao Tung University, Hsinchu 300, Taiwan.*

[†]*Both authors contributed equally to this work*

E-mail: c00jsw00@kmu.edu.tw, d830166@kmu.edu.tw, yhchan@nycu.edu.tw, linhc@nycu.edu.tw

Table of Contents

Table of Contents……………………………………………………………………S2

Experimental procedures…………………………………………………………...S3

1. **Materials……………………………………………………………………… S3**
2. **Preparation of T2-02 coacervates…………………………………………… S3**
3. **Preparation of T2-02 coacervate NPs…………………………………………S3**
4. **Determination of calibration curves for T2-02 AMP……………………….....S3**

Physiochemical Characterization…………………………………………………… S3

Figure S1 UV-VIS absorption spectra and calibration curve of T2-02……….....S4

Figure S2- S4 Particle size distribution of Lipo nanoparticles …………..……….....S5

Figure S5- S6 Particle size distribution of Coacervates nanoparticles ……………..S7

Figure S7- S8 Zeta Potential for coacervate nanoparticles (DLS)…………………..S8

Figure S9 Zeta Potential for Lipo nanoparticles (DLS)…………………………S9

Figure S10 Effect of salt on coacervates…………………………………………...S9

Scheme S1 Schematic of T2-02 Coacervate nanoparticles……………………....S10

Tabulations

Table S1- S3 Formulation Optimization and Cytotoxicity………………………...S10

Table S4- S6 Antimicrobial Activity…….………………………………………..S11

Experimental Procedures

(1) Materials

All chemicals and reagents used were of analytical grade. The T2-02 peptide (MW 2646.27 Da) was custom-synthesized by Kelowna International Scientific Inc, Lauric acid sodium salt (LA, MW 222.30 Da) was obtained from Tokyo Chemical Industry Co., Ltd., 1,2-Dimyristoyl-sn-glycero-3-phosphoethanolamine-N-[methoxy(polyethylene glycol)-2000] (ammonium salt) (DSPE-PEG_{2000}, MW 2693.285 Da) was purchased from Laysan Bio Inc., 1,2-Dioleoyl-sn-glycero-3-phosphocholine (DOPC, MW 786.1 Da) was acquired from BroadPharm, and Poly-L-lysine hydrobromide (PLL, MW 30-70 kDa) was sourced from Sigma Aldrich.

(2) Preparation of T2-02 Coacervates

A solution of cationic T2-02 and anionic lauric acid (LA) was prepared at an 8:2 mass concentration ratio by separately dissolving 1.1 mg of T2-02 and 0.275 mg of LA in 135.5 μL of deionized (DI) water each. The two solutions were then mixed in a 1:1 volume ratio, combining 135.5 μL of the T2-02 solution with 135.5 μL of the LA solution. Upon mixing, the oppositely charged polyelectrolytes interacted, resulting in the immediate formation of T2-02/LA coacervate droplets, which caused the solution to become turbid.

(3) Preparation of T2-02 Coacervate NPs

The T2-02 coacervate nanoparticles were prepared using an optimized film-rehydration method. First, a suspension of positively charged T2-02/LA coacervate droplets, with an 8:2 ratio, was coarsened for 30 minutes and stored for subsequent steps. Phospholipids DOPC and DSPE-PEG2000, in a 1:1 ratio, were dissolved in chloroform and transferred to a round-bottomed flask. The mixture was then subjected to vacuum rotary evaporation for 30 minutes. Next, the coacervate droplet suspension was slowly added to the flask and hydrated for 1 hour, ensuring a 1:1 mass ratio of phospholipids to polyelectrolytes. The hydrated suspension was carefully mixed with a pipette and transferred to a centrifuge tube, where it was allowed to settle for 2 hours. The suspension was then centrifuged at 10,000 rpm for 10 minutes, resuspended in PBS buffer, and finally treated with a PLL solution to complete the assembly of T2-02 coacervate NPs.

(4) Determination of Calibration curves for T2-02 AMP

Calibration curves for T2-02 were generated by measuring the UV-VIS absorbance of T2-02 in deionized (DI) water at known concentrations (Figure S1A). The absorbance values were plotted against the corresponding T2-02 concentrations to create a calibration curve (Figure S1B). This curve was then utilized to determine the T2-02 concentration in samples after

centrifuging the liposomal suspension. The resulting data provided valuable information on the encapsulation efficiency of T2-02 within the liposomes.

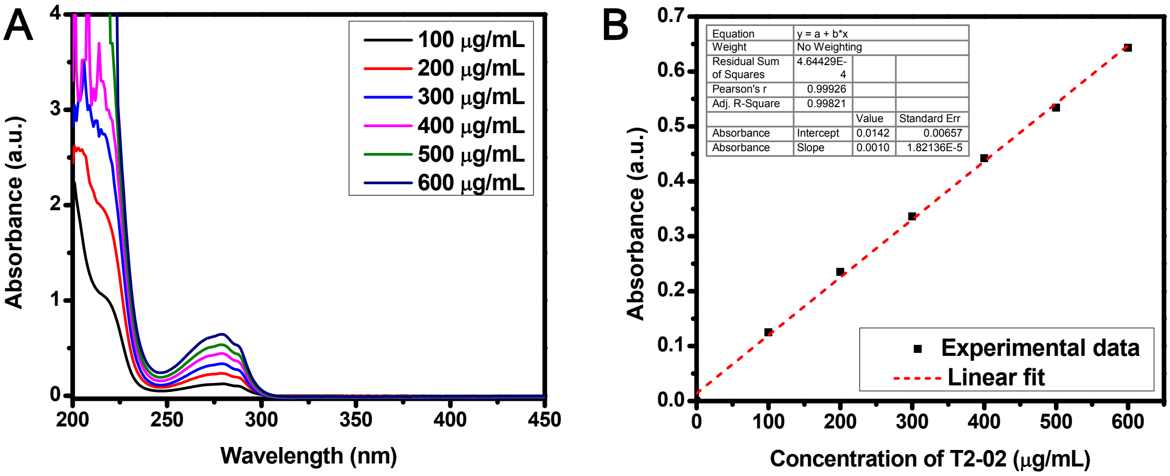

Figure S1. (A) UV-VIS absorption spectra of aqueous T2-02 solutions at various concentrations used to determine the (B) calibration curve of T2-02 in water.

Determined calibration curve:

$$C(T2-02) = \frac{A-0.014}{0.001} \text{ ----------------- (Eq. S1)}$$

Where A is the absorbance at 280 nm determined from UV-VIS absorption spectra, C(T2-02) is the concentration of T2-02 AMP encapsulated in the liposomal suspension.

Physiochemical Characterization

Particle size characterizations

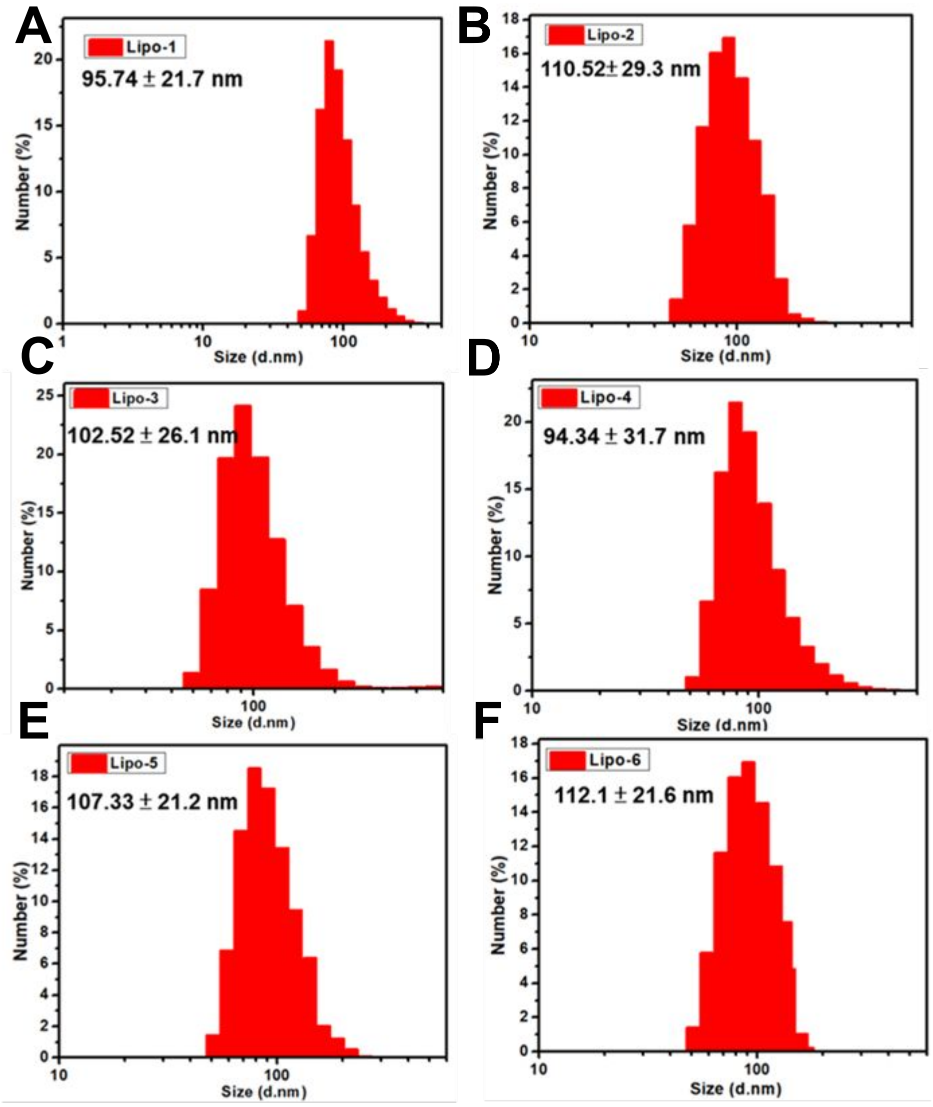

Figure S2. Particle size distribution of (A) Lipo-1, (B) Lipo-2, (C) Lipo-3, (D) Lipo-4, (E) Lipo-5, and (F) Lipo-6 nanoparticles measured using DLS. Statistical analyses were performed with a sample size of three (*n* = 3)

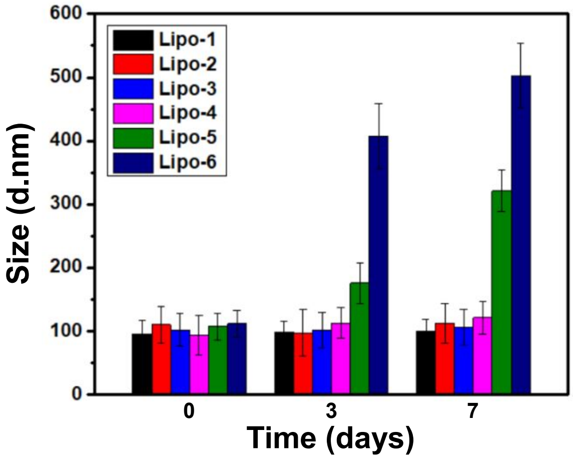

Figure S3. Particle size analysis indicating the colloidal stability of Lipo- (1 to 6) nanoparticles with (w) and without (w/o) PLL coating. Statistical analyses were performed with a sample size of three (n = 3).

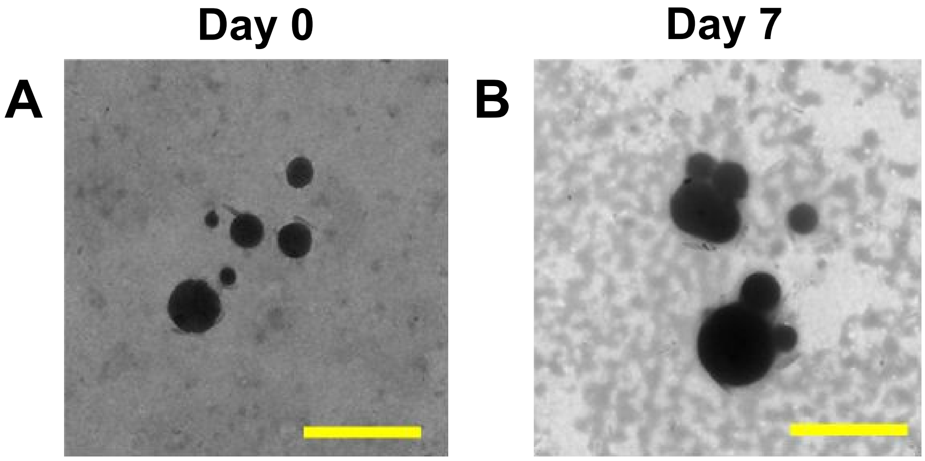

Figure S4. Transmission Electron Microscopy (TEM) images of Lipo-6 nanoparticle i.e. w/o PLL coating showing aggregation effects measured at (A) Day 0 and (B) Day 7. (scale bar: 500 nm).

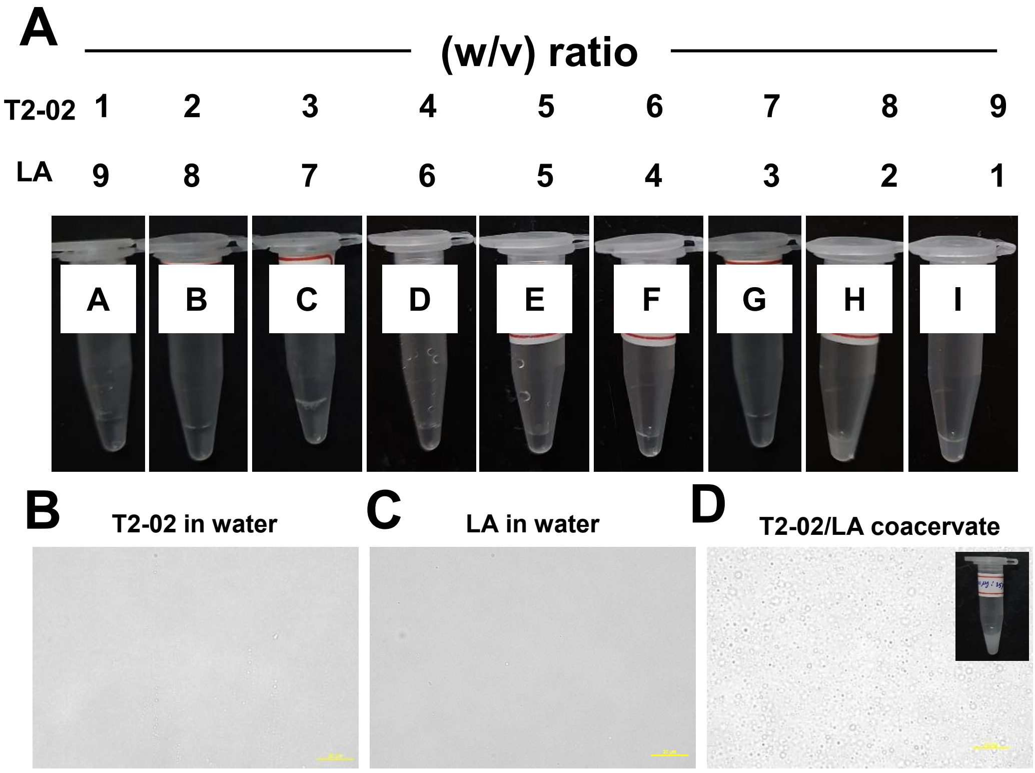

Figure S5. A.) Coacervate complexation between T2-02 AMP and LA at different weight ratios. Increasing the weight percent of T2-02 beyond 70% in the T2-02/LA mixture led to visible cloudiness indicative of coacervate complexation. Coacervate droplet imaging using optical microscope. (B) T2-02 in water, (C) LA in water as control and (D) T2-02 coacervates at a T2-02 to LA weight ratio of 8:2. (Scale bar: 20 μm)

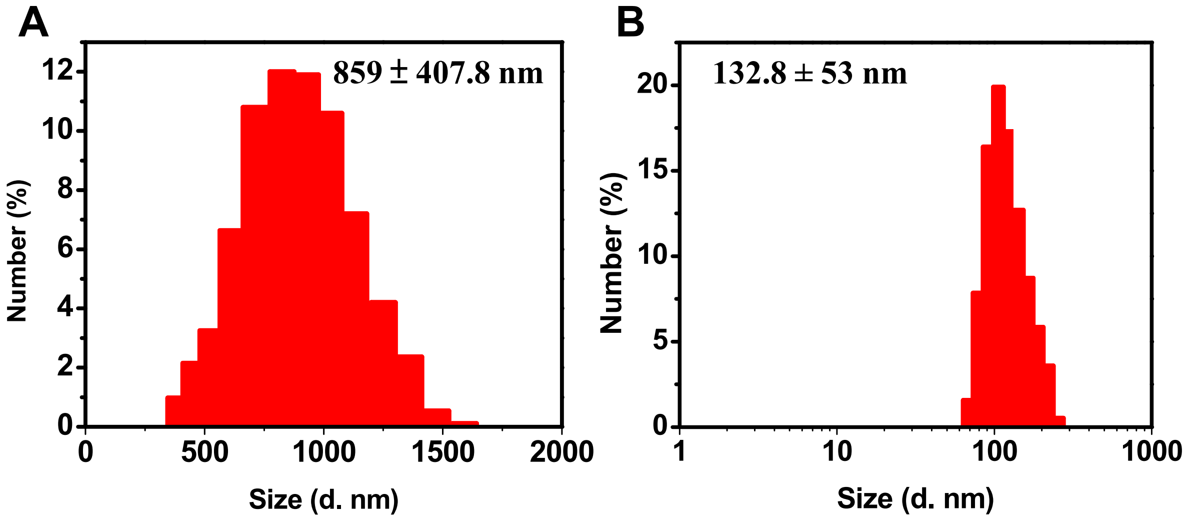

Figure S6. Particle size distribution of (A) T2-02 coacervate and (B) T2-02 coacervate NPs measured using DLS. (n=3)

Zeta potential measurements of coacervates and liposomal nanoparticles

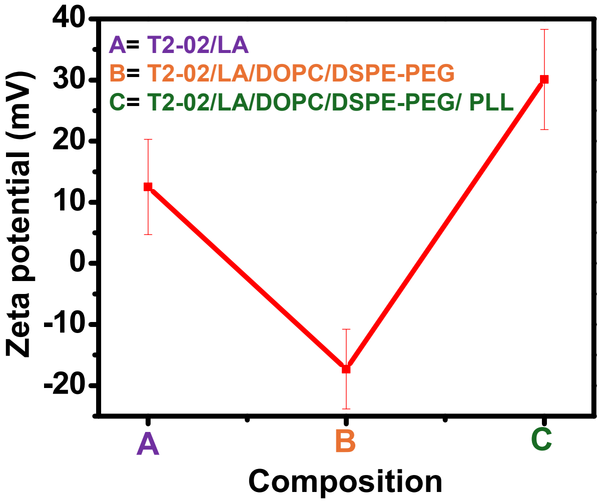

Figure S7. Zeta potential measurements demonstrating charge reversal upon T2-02 coacervate nanoparticles formation. (n= 3)

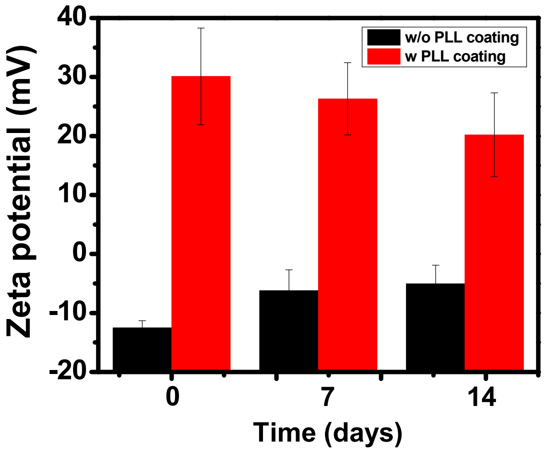

Figure S8. Zeta potential analysis indicating the colloidal stability of T2-02 Lipo nanoparticles with (w) and without (w/o) PLL coating. Statistical analyses were performed with a sample size of three (n = 3) measured at 25°C.

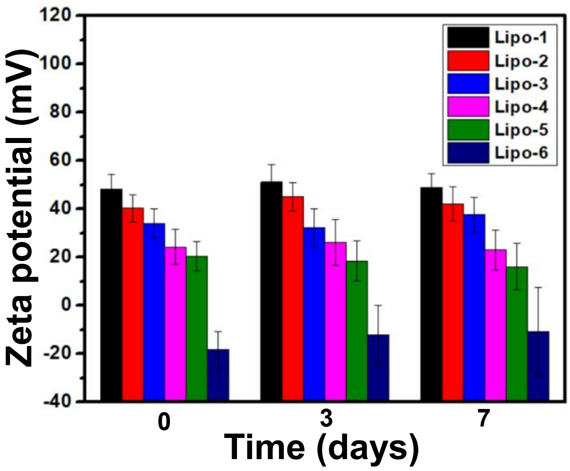

Figure S9. Zeta potential analysis indicating the colloidal stability of T2-02 Lipo nanoparticles with (w) and without (w/o) PLL coating. Statistical analyses were performed with a sample size of three (n = 3) measured at 25°C.

Salt Induced Aggregation of Coacervates

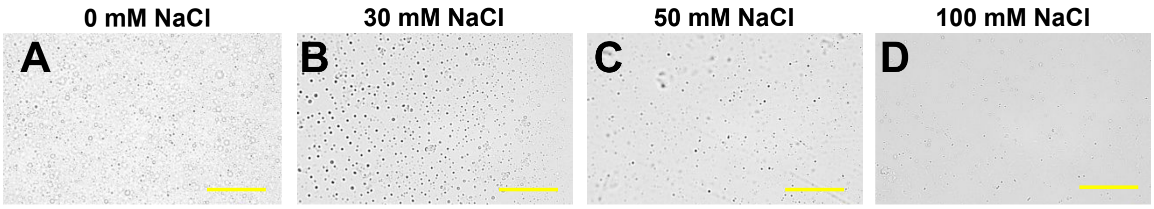

Figure S10. Effect of Salt concentration with (A) 0 mM, (B) 30 mM, (C) 50 mM, and (D) 100 mM NaCl solutions on T2-02 Coacervates prepared from 8:2 ratio of T2-02/LA (Scale bar: 20 μm)

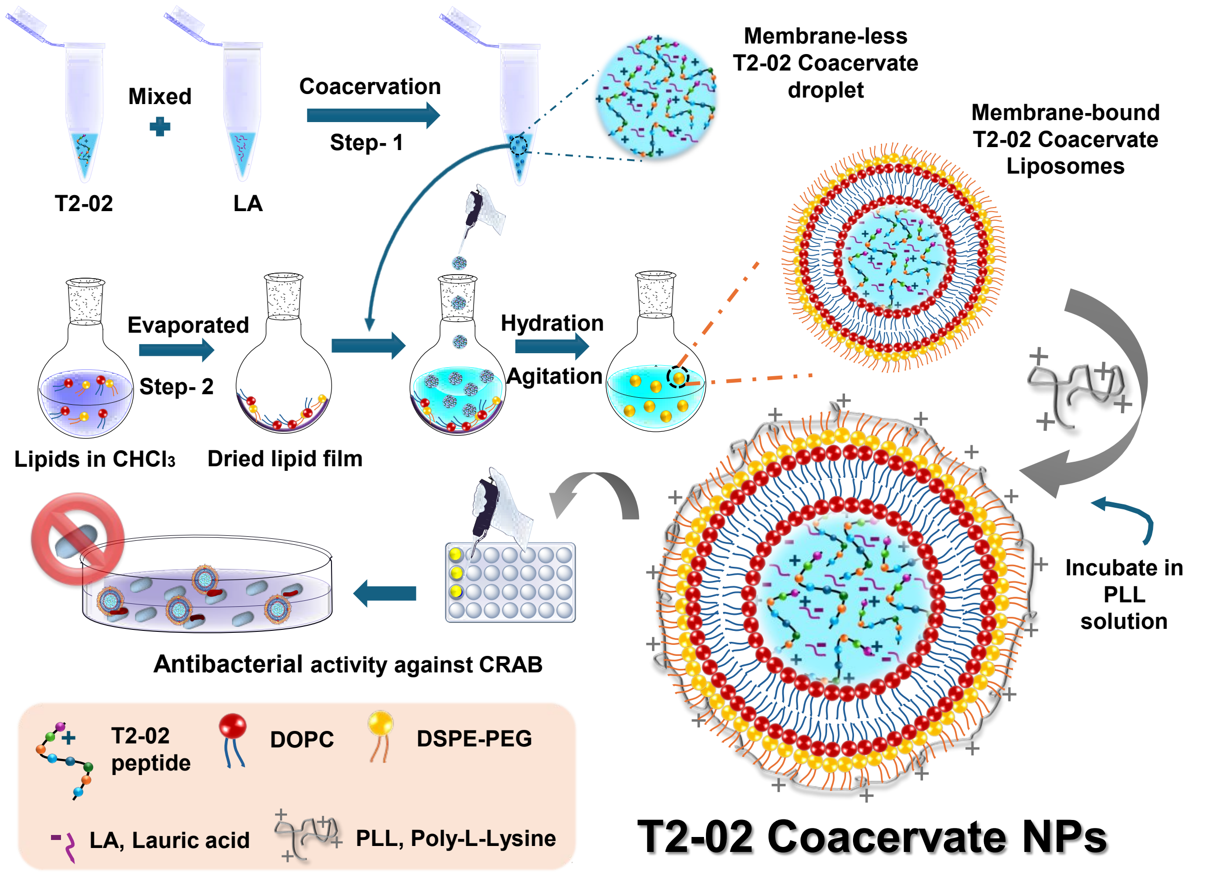

Scheme S1. Schematic representation of design of T2-02 Coacervate NPs against CRAB.

Tabulations

Formulation Optimization and Cytotoxicity

Table S1. Optimisation of ratio of T2-02 to lipids to Poly-L-Lysine (PLL) and their particle characteristics and cytotoxicity evaluation.

Label	T2-02 (mg)	DOPC (mg)	DSPE-PEG_{2000} (mg)	PLL (mg)	Particle Size (d.nm) (Number)	Zeta potential (mV)	Final conc. of T2-02 (μg/ml)	Encapsulation efficiency (%)	IC_{50} value HK-2 cell (μg/ml)
**Lipo- 1**	2.2	1.1	1.1	1	95.74 ± 21.7	+48.1± 6.1	366.66	39	23
**Lipo- 2**	2.2	1.1	1.1	0.1	110.52±29.3	+40.4± 5.61	366.66	41	270
**Lipo- 3**	2.2	1.1	1.1	0.055	102.52±26.1	+34.1± 6.12	733.33	50.55	514
**Lipo- 4**	2.2	1.1	1.1	0.0275	94.34 ± 31.7	+24.3± 7.31	733.33	48.25	256
**Lipo- 5**	2.2	1.1	1.1	0.0055	107.33±21.2	+20.4± 6.1	733.33	49	197
**Lipo- 6**	2.2	1.1	1.1	0	112.11±21.6	-18.4± 2.7	733.33	52.31	154
**T2-02 Lipo**	4.4	1.1	1.1	0.055	131.9±55	+32.1±6.21	5580	63.41	819.62

Table S2. Determining the encapsulation efficiency of T2-02 in T2-02 Lipo using centrifugation and UV-Vis spectroscopy.

Sample	Wavelength (nm)	Absorbance of samples	Absorbance (residue) Ar = Am – As	Absorbance (supernatant) As = Am – Ar	C(T2-02)= $\frac{A+0.029}{0.001}$ (µg/mL)
**Mixture (m)**	280	0.4746	**0.2934**	0.1583	279 µg/mL*
**Supernatant (s)**	280	0.1812			
**Residue (r)**	280	0.3163			

*100 µL of residue contains 279 µg/mL of T2-02 encapsulated. Therefore, 1 mL of initial formulation will contain 2790 µg/mL. The bulk residue is diluted to a volume of 0.5 mL to get the higher concentration of 5580 µg/mL.

Table S3. Optimisation of ratio of T2-02 coacervate to lipids to PLL and their particle characteristics and cytotoxicity evaluation.

Label	T2-02 (mg)	LA (mg)	DOPC (mg)	DSPE-PEG_{2000} (mg)	PLL (mg)	Particle Size (d.nm) (Number)	Zeta potential (mV)	Final conc. of T2-02 (µg/ml)	IC_{50} value HK-2 cell (µg/ml)
**T2-02 coacervate**	1.1	0.275	-	-	-	859 ± 407	+12.5±7.8	4000	NA
**T2-02 coacervate NPs**	1.1	0.275	1.1	1.1	1	132.8±53	+30.1±8.2	4000	15

Antimicrobial Activity

Table S4. MICs of antimicrobial compounds against *A. baumanni*i ATCC19606 in the presence of divalent cations.

Antimicrobial Compounds	Antibacterial activity (MIC) (µg/ml)	
	Control	20 mM $CaCl_2$
**Colistin**	2	32
**T2-02**	8	>256
**T2-02 Lipo**	8	>256

Table S5. MICs of various T2-02 liposomal formulations.

CRAB Isolates	Free T2-02 (µg/ml)	Lipo-1 (µg/ml)	Lipo-2 (µg/ml)	Lipo-3 (µg/ml)
**AB-01**	8	8	8	8
**AB-02**	8	2	2	4
**AB-03**	4	2	2	8
**AB-04**	8	2	2	8
**AB-05**	8	2	2	4
**AB-06**	8	2	2	8
**AB-07**	8	2	2	2
**AB-08**	8	8	8	16

Table S6. MICs of various T2-02 coacervate formulations.

CRAB Isolates	Free T2-02 (µg/ml)	T2-02 coacervate (µg/ml)	T2-02 coacervate NPs (µg/ml)
**AB-01**	8	>32	8
**AB-02**	8	>32	8
**AB-03**	4	>32	4
**AB-04**	8	>32	2
**AB-05**	8	>32	8
**AB-06**	8	>32	8
**AB-07**	8	>32	4
**AB-08**	8	>32	8
**AB-09**	8	>32	8
**AB-10**	8	>32	8
**AB-11**	8	>32	8
**AB-12**	16	>32	4
**AB-13**	4	>32	2
**AB-14**	8		8
**AB-15**	8		4
**AB-16**	16		8
**AB-17**	8		8
**AB-18**	16		8
**AB-19**	8		4
**AB-20**	8		2
**AB-21**	8		8
**AB-22**	8		8
**AB-23**	8		4
**AB-24**	8		8
